# Study on association of working hours and occupational physical activity with the occurrence of coronary heart disease in a Chinese population

**DOI:** 10.1371/journal.pone.0185598

**Published:** 2017-10-19

**Authors:** Yao Ma, Ying-Jun Wang, Bing-Rui Chen, Hao-Jie Shi, Hao Wang, Mohammad Reeaze Khurwolah, Ya-Fei Li, Zhi-Yong Xie, Yang Yang, Lian-Sheng Wang

**Affiliations:** 1 Department of Cardiology, the First Affiliated Hospital of Nanjing Medical University, Nanjing, China; 2 Division of Cardiology, Sheyang County People’s Hospital, Yancheng, China; University of Bologna, ITALY

## Abstract

**Objective:**

To explore the association of working hours and occupational physical activity (OPA) with the occurrence of coronary heart disease (CHD) in a Chinese population.

**Methods:**

A total of 595 participants (354 and 241 patients with and without CHD, respectively) aged between 24 and 65 were enrolled in our study, which was conducted at the First Affiliated Hospital of Nanjing Medical University between December 2015 and October 2016. Participant characteristics were collected from face-to-face questionnaires, and logistic regression analysis was conducted to examine the association of working hours and OPA with the occurrence of CHD.

**Results:**

Compared with non-employed people, long working hours (especially ≥55 hours/week) contributed to the occurrence of CHD (adjusted odds ratio[OR] = 2.213, 95% confidence interval [CI]: 1.125, 4.355, P = 0.021) after multivariate adjustment in the Chinese population. With the extension of worktime, the CHD risk increased (P for the dose-response trend = 0.022). Meanwhile, even after adjusting for engagement in physical activity during leisure time, sedentary behavior at work had an adverse effect on CHD risk (adjusted OR = 2.794, 95%CI: 1.526, 5.115, P = 0.001), and a linear relationship was also found between OPA and CHD (P for the trend = 0.005).

**Conclusions:**

Long working hours and sedentary behavior at work are associated with a high risk of CHD. In addition, prolonged working hours in sedentary occupations increases the risk of CHD, independent of engagement in leisure time physical activity.

## Introduction

Coronary heart disease (CHD) remains the leading cause of death and disability across the world[1]. During the past few decades, there has been an increased focus on investigating the relationship between psycho-social work factors and CHD. Related studies have revealed that job stress[2], overtime work[3, 4], job insecurity[5, 6] and occupational physical activity (OPA)[7, 8] are linked with CHD.

Long working hours and sedentary jobs have become a major component of work patterns around the world. The International Labor Organization (ILO) has reported that 22.0% of workers globally are working over the standard recommended working hours, usually>48 hours per week[9]. In 2004–2005, the percentage of workers who worked ≥49 hours per week was 49.5% in Korea, 23.6% in New Zealand, 20.4% in Australia and 18.1% in the US. A similar trend of increasing work hours per week has also been reported in China[10]. As technology continues to develop, work has become more mentally demanding, which in turn has led to increased sedentariness. The majority of the workforce spends more than 70% of their working time sitting[11]. Epidemiological data have shown that OPA has decreased in recent decades around the world. In America, almost 50% of industry occupations required at least moderately intense OPA in the early 1960s, whereas currently, less than 20% of industry occupations have this requirement. These changes have caused daily occupation-related energy expenditure to decrease by more than 100 calories[12]. Similarly, sedentary behavior and low OPA level in the workplace are prevalent in China. Over the period 1991–2006 in China, the average level of physical activity among adults declined by about 32%[13].

Despite extensive investigation, the relationship between working hours, OPA and health remains poorly understood. Several reports have indicated that long working hours negatively affect health and increase CHD risk[3, 14, 15], whereas other investigators have claimed that short working hours increase the risk of acute myocardial infarction (AMI)[16]. Furthermore, some studies have concluded that the incidence of CHD and all-cause mortality rose with increased OPA[17–19], whereas other studies have shown the opposite result, namely, that engagement in little OPA or sedentary work is detrimental to health[18–20]. Although some researchers advocate that sedentary workers increase their leisure-time physical exercise, this does not offset the harm that results from sitting for long hours at work. At present, little is known about the association between working hours, OPA and CHD risk in mainland China. Therefore, the purpose of this study was to investigate the relationship between working hours and OPA with the occurrence of CHD in a Chinese population.

## Methods

### Study design and participants

This study enrolled 595 participants aged between 24 and 65 during the period of December 2015 to November 2016 in Nanjing, China. All subjects underwent coronary angiography (CAG) for the first time in the First Affiliated Hospital of Nanjing Medical University due to chest pain, or abnormal electrocardiogram. The CHD group included 354 subjects who had at least one main coronary artery (left main trunk, left anterior descending coronary artery, left circumflex coronary artery or right coronary artery) with >50% luminal diameter stenosis found on CAG, and were first diagnosed with CHD. The non-CHD group consisted of 241 subjects who were free of CHD as per diagnostic CAG. Patients with long-standing chronic disease such as chronic kidney disease, chronic obstructive pulmonary disease, heart failure, malignant tumors, cardiomyopathy or myocarditis were excluded from the study as these could influence the choice of career. Patients who had undergone CAG and were diagnosed with CHD before the study were excluded, as were laid-off workers.

Employed people were defined as having a job that paid enough to support daily living[21]. This included employees who were not working because of vacation, a business trip, sick leave or other reasons enlisted in the questionnaire. Non-employment was defined as being economically inactive or unemployed[22] and included individuals who reported no jobs and depended on government pension.

### Approvals and patient consent

The study was approved by the Ethics Committee of the First Affiliated Hospital of Nanjing Medical University (Nanjing, China). Written informed consent for the purpose of this research was obtained from each participant.

### Questionnaire

#### Measurement of working hours

All the participants completed the questionnaire regarding working hours and other working characteristics before undergoing CAG. They were all asked the question “how much time did you usually spend working per week at your primary job before being hospitalized?”. Based on their answers, we divided the participants into several groups based on working time per week as follows: <35 h, 35–40 h, 41–48 h, 49–54 h, and ≥55 h. The definition of long working hours has varied among previous related studies, and in agreement with a meta-analysis in the Lancet, we defined long working hours as being 41 h or more per week[23].

#### Classification of occupational physical activity

Our evaluation of work-related physical activity was based on an occupational physical activity questionnaire (OPAQ)[24] containing seven questions (S1 Appendix). Question 1 addressed working hours per week, and questions 2 to 7 focused on the amount of OPA. According to their answers, we divided the participants into 4 OPA levels: sedentary behavior, usually referred to as sitting office work; light OPA, defined as mostly standing; moderate OPA, defined as mostly walking; and heavy OPA, defined as heavy manual labor. Each type of work had a corresponding metabolic equivalent task (MET): sedentary behavior had an energy expenditure less than 1.5 METs, light OPA between 1.6 and 3.0 METs, moderate OPA between 3.1 and 4.5 METs, and heavy OPA >4.5 METs[25–27].

### Data collection

All data were collected by trained interviewers using a well-designed questionnaire before CAG. Age, body mass index (BMI), gender, hypertension, diabetes, hyperlipidemia, family history of CHD (any kind of CHD in first-degree relatives), level of education, and lifestyle behaviors such as sports-related physical activity, smoking, drinking and employment status were included in the questionnaire. Age and BMI were considered as continuous variables and were normally distributed. Blood pressure, total cholesterol (TC), high-density lipoprotein (HDL), low-density lipoprotein (LDL), and fasting blood glucose levels were measured for every participant during their hospitalization.

Hypertension was diagnosed as having a history of hypertension, receiving antihypertensive therapy, or newly diagnosed with hypertension with two blood pressure readings higher than 140 mmHg systolic and/or 90 mmHg diastolic. Diabetes mellitus (DM) was defined as a fasting plasma glucose >126 mg/dL (7.0 mmol/L) or a 2-h plasma glucose >200 mg/dL (11.1 mmol/L) during an oral glucose tolerance test, or a random plasma glucose >200 mg/dL (11.1 mmol/L), or under hypoglycemic treatment. Hyperlipidemia was defined as a serum concentration of TC >200 mg/dL (5.18 mmol/L), TG >150 mg/dL (1.7 mmol/L), LDL >130 mg/dL (3.37 mmol/L), or HDL <40 mg/dL (1.04 mmol/L) in men and <50 mg/dL (1.30 mmol/L) in women, or receiving dyslipidemia treatment[28].

Sports-related physical activity, described as exercising regularly during leisure time, was defined as exercising for more than 150 min/week with a minimal intensity of 3 METs for at least 5 years, including in the 3 months prior to hospitalization[29].

### Psychosocial work factors

Job strain was assessed using the Job Content Questionnaire (JCQ) Scales[30], which contain 11 items on job control (job decision authority and skill discretion) and job demand (working hard and fast with insufficient time to accomplish job tasks) (S2 Appendix). Every item had a score of 0–10, and job strain was divided into 3 categories: low, moderate and high. Low job strain was defined as a score below the median on job demand and a score above the median on decision latitude. High job strain was defined as high psychological demand and low decision latitude. Moderate job strain was defined as high demand and high control, or as low demand and low control. Job security was evaluated in the JCQ scales using the statement “My job is secure”[31]. Based on the response to this statement (strongly agree, agree, disagree, and strongly disagree), we characterized the participants as having answered “yes” (strongly agree, agree) or “no” (disagree, strongly disagree). Rest time was defined as days off of work per month.

### Statistical analysis

All data were processed using Statistics Package for Social Sciences (ver. 16.0; SPSS Incorporated, Chicago, IL, USA). Age and BMI were considered continuous variables and were presented as the mean±SD; they were evaluated by variance analysis. The other characteristics were considered categorical variables and evaluated by chi-squared test. Univariate and multiple logistic regression analyses were used to calculate odds ratios (ORs) and 95% confidence intervals (CIs) to exclude the influences of confounding variables. For subgroup analysis, non-employed subjects were regarded as the reference group. ORs and 95% CIs were calculated, and a linear trend test was used to reveal the associations between the incidence of CHD and different occupational features. Differences were considered significant at a P-value <0.05 and all P-values were 2-tailed.

## Results

### Baseline characteristics

Table 1 shows the baseline characteristics of all the participants in the study. The average age in the CHD group was higher than that in the control group, and the difference was statistically significant (P<0.001). Similarly, the mean BMI was higher in the CHD group than in the control group (P = 0.011). Overall, 77.4% of participants in the CHD group were male; in the control group, 54.8% were male. The prevalence of hypertension, diabetes, hyperlipidemia in the CHD group (70.6%, 26.8%, and 71.2%) was higher compared to that in the control group (52.7%, 8.7%, and 56.0%), and the differences were all statistically significant (P<0.001). In the CHD group, the proportion of participants with a family history of CHD (22.6%) was significantly higher than in the control group (12.9%). The CHD group also had a greater number of participants who were current smokers or drinkers compared to the control group. However, the percentage of participants engaging in sports-related physical activity was lower in the CHD group (15.0%) than control group (22.0%). The level of education was similar between the two groups. As for employment status, 85.6% and 79.3% of the participants in the CHD group and control group were employed, respectively, and this difference was statistically significant (P = 0.043).

**Table 1:** Characteristics of CHD and non-CHD groups.

Characteristics	CHD(%)(n = 354)	Non-CHD(%)(n = 241)	P
Age	55.28±7.01	51.94±8.55	<0.001
Male	274(77.4)	132(54.8)	<0.001
BMI	25.54±2.91	24.90±3.18	0.011
Hypertension	250(70.6)	127(52.7)	<0.001
Diabetes	95(26.8)	21(8.7)	<0.001
Hyperlipidemia	252(71.2)	135(56.0)	<0.001
Family history of CHD	80(22.6)	31(12.9)	0.003
Sports-related Physical activity	53(15.0)	53(22.0)	0.028
Smoking status			<0.001
Never	146(41.2)	146(60.6)	
Former	52(14.7)	23(9.5)	
Current	156(44.1)	72(29.9)	
Drinking status			0.01
Never	177(50.0)	160(66.4)	
Former	70(19.8)	30(12.4)	
Current	107(30.2)	51(21.2)	
Education			0.249
Illiteracy	18(5.1)	17(7.1)	
Primary	54(15.3)	26(10.8)	
Middle	98(27.7)	81(33.6)	
High	113(31.9)	68(28.2)	
College	71(20.1)	49(20.3)	
Employment status	303(85.6)	191(79.3)	0.043

Abbreviations: CHD, coronary heart disease; BMI, body mass index.

Continuous values (age and BMI) were expressed as mean ± SD and Student’s t-test was used for comparison.

The rest were categorical variables expressed as numbers and frequencies (%), compared by Pearson’s χ2-test.

The results of univariate logistic regression analysis (Table 2) indicated that participants who were older, had a higher BMI, were male, and who smoked or consumed alcohol were more likely to have CHD. Moreover, the participants with CHD had a higher prevalence of hypertension, diabetes, hyperlipidemia and family history of CHD. Exercise acted as a protective factor, whereas employment status acted as a risk factor.

**Table 2:** Univariate logistic regression for CHD risk factors.

Characteristic	All subjects		
	OR	95%CI	P
Age	1.057	1.034, 1.081	<0.001
Gender (Male = 1, Female = 2)	0.354	0.248, 0.505	<0.001
BMI	1.073	1.016, 1.134	0.012
Hypertension (N = 0, Y = 1)	2.196	1.563, 3.086	<0.001
Diabetes (N = 0, Y = 1)	3.843	2.318, 6.371	<0.001
Hyperlipidemia (N = 0, Y = 1)	1.940	1.377, 2.733	<0.001
Family history of CHD (N = 0, Y = 1)	1.978	1.259, 3.108	0.003
Sports-related Physical activity (N = 0, Y = 1)	0.625	0.410, 0.953	0.029
Smoking status			
Never	1		
Former	2.261	1.315, 3.887	0.003
Current	2.167	1.510, 3.110	<0.001
Drinking status			
Never	1		
Former	2.109	1.308, 3.402	0.002
Current	1.897	1.276, 2.818	0.002
Education			
Illiteracy	1		
Primary	1.962	0.871, 4.415	0.104
Middle	1.143	0.553, 2.360	0.719
High	1.569	0.758, 3.250	0.225
College	1.368	0.642, 2.915	0.416
Employment status (N = 0, Y = 1)	1.555	1.012, 2.391	0.044

Abbreviations: CHD, coronary heart disease; BMI, body mass index; OR, odds ratio; CI, confidence interval.

Table 3 shows the results of multivariate logistic regression analysis comparing the CHD patients and controls. The analysis revealed significant differences in age (OR: 1.059, 95% CI: 1.032, 1.087, P<0.001), gender (OR: 0.324, 95% CI: 0.186, 0.565, P<0.001), hypertension (OR: 1.773, 95% CI: 1.198, 2.624 P = 0.004), diabetes (OR: 3.447, 95% CI: 1.985, 5.986, P<0.001), hyperlipidemia (OR: 1.627, 95% CI: 1.090, 2.431, P = 0.017) and family history of CHD (OR: 1.878, 95% CI: 1.126, 3.130, P = 0.016). Exercise (OR: 0.585, 95% CI: 0.361, 0.949, P = 0.03) was a protective factor against CHD. There were no statistically significant differences related to employment status, BMI, education, or smoking and drinking status between the CHD patients and controls.

**Table 3:** Multivariate logistic regression for CHD risk factors.

Characteristic	All subjects		
	OR	95%CI	P
Age	1.059	1.032, 1.087	<0.001
Gender (Male = 1, Female = 2)	0.324	0.186, 0.565	<0.001
BMI	1.053	0.989, 1.123	0.109
Hypertension (N = 0, Y = 1)	1.773	1.198, 2.624	0.004
Diabetes (N = 0, Y = 1)	3.447	1.985, 5.986	<0.001
Hyperlipidemia (N = 0, Y = 1)	1.627	1.090, 2.431	0.017
Family history of CHD (N = 0, Y = 1)	1.878	1.126, 3.130	0.016
Sports-related Physical activity (N = 0, Y = 1)	0.585	0.361, 0.949	0.030
Smoking status			
Never	1		
Former	1.026	0.507, 2.077	0.944
Current	1.178	0.685, 2.025	0.554
Drinking status			
Never	1		
Former	1.216	0.677, 2.184	0.513
Current	1.010	0.602, 1.696	0.970
Education			
Illiteracy	1		
Primary	2.002	0.798, 5.024	0.139
Middle	0.864	0.372, 2.003	0.732
High	0.966	0.405, 2.302	0.938
College	0.839	0.338, 2.082	0.705
Employment status (N = 0, Y = 1)	1.396	0.834, 2.337	0.204

Abbreviations: CHD, coronary heart disease; BMI, body mass index; OR, odds ratio; CI, confidence interval.

Multivariate adjustment for age, gender, body mass index, hypertension, diabetes mellitus, hyperlipidemia, smoking status, alcohol use, physical activity, and education.

### Occupational characteristics and CHD

Table 4 illustrates the relationships found between occupational characteristics and CHD. The proportion of participants under high job strain in the CHD group (33.1%) was significantly higher than that in the control group (17%). After multivariate adjustment, job strain was a risk factor (P for the linear trend = 0.004) for CHD, and high job strain was considered to result in high CHD risk (adjusted OR = 2.384, 95%CI: 1.290, 4.405, P = 0.006). However, the distributions of job security and rest time per month were similar between the CHD and control groups. Thus, there was no obvious significant difference in the dose-response trends between job security and rest time per month between the two groups.

**Table 4:** Relationship between occupational characteristics and CHD.

Characteristics	CHD (n = 354)	Non-CHD (n = 241)	Unadjusted OR(95%CI)	P	Adjusted^b^ OR(95%CI)	P
Employment status^a^						
NO	51	50	1		1	
Yes	303	191	1.555(1.012, 2.391)	0.044	1.396(0.834, 2.337)	0.204
Job Strain^a^						
None	51(14.4)	50(20.7)	1		1	
Low	63(17.8)	54(22.4)	1.144(0.671, 1.949)	0.621	1.054(0.571, 1.944)	0.867
Moderate	123(34.7)	96(39.8)	1.256(0.783, 2.015)	0.344	1.205(0.690, 2.104)	0.513
High	117(33.1)	41(17.0)	2.798(1.650, 4.743)	<0.001	2.384(1.290, 4.405)	0.006
P for trend				<0.001		0.004
Rest time^a^						
None	51(14.4)	50(20.7)	1		1	
≤4 days/month	194(54.8)	128(53.1)	1.486(0.948, 2.329)	0.084	1.419(0.830, 2.427)	0.201
5–8 days/month	104(29.4)	56(23.2)	1.821(1.096, 3.025)	0.021	1.505(0.819, 2.764)	0.188
>8 days/month	5(1.4)	7(2.9)	0.700(0.208, 2.353)	0.565	0.479(0.122, 1.882)	0.291
P for trend				0.108		0.613
Security of work^a^						
None	51(14.4)	50(20.7)	1		1	
Insecurity	61(17.2)	36(14.9)	1.661 (0.942, 2.930)	0.080	1.669(0.867, 3.212)	0.125
Security	242(68.4)	155(64.3)	1.531 (0.987, 2.375)	0.057	1.326(0.782, 2.249)	0.294

Abbreviations: CHD, coronary heart disease; OR, odds ratio; CI, confidence interval.

^a^ Qualitative variables were used to express as numbers and frequencies (%) tested by Pearson’s χ2, and both were statistically significant (P<0.05).

^b^ Adjustment for age, gender, body mass index, hypertension, diabetes mellitus, hyperlipidemia, smoking status, alcohol use, physical activity, and education.

### Worktime, OPA and CHD risk

Table 5 presents the identified associations between worktime, OPA and the incidence of CHD. The majority of the employed participants (56.9%) worked overtime (>40 h/w). A higher proportion of participants engaged in long working hours in CHD group (53.1%) than control group (38.6%), and a significant difference (P<0.05) was found in the number of participants working ≥ 55 h/w between the CHD (20.3%) and control groups (10.8%). Compared to the non-employed group, the incidence of CHD increased with the extension of working hours, and the dose-response for the trend was statistically significant (adjusted P = 0.022). Working more than 55 h/w was associated with an increased risk of CHD with a crude OR of 2.715 (95% CI: 1.498, 4.919, P = 0.001) and an adjusted OR of 2.213 (95% CI: 1.125, 4.355, P = 0.021) after multivariate adjustment for age, gender, hypertension, diabetes, hyperlipidemia, family history of CHD and sports-related physical activity. Sedentary behavior at work accounted for nearly half of the participants in the CHD group (44.4%) as opposed to 22.4% in the control group, and the difference was statistically significant. We also found that a sedentary occupation was a risk factor for CHD, even after adjusting for engagement in physical activity at leisure time (adjusted OR = 2.794, 95%CI: 1.526, 5.115, P = 0.001). A significant linear relationship was observed between OPA and CHD after multivariate adjustment (P for the trend = 0.005), and the lesser the OPA during working, the higher the incidence of CHD. Therefore, the participants who worked prolonged hours at sedentary occupations had an increased risk of CHD.

**Table 5:** Relationship between worktime, OPA and the risk of CHD.

Characteristics	CHD (n = 354)	Non-CHD (n = 241)	Unadjusted OR(95%CI)	P	Adjusted ^b^ OR(95%CI)	P
Worktime^a^						
None	51(14.4)	50(20.7)	1		1	
<35 hours/week	25(7.1)	12(5.0)	2.042(0.926, 4.506)	0.077	1.825(0.739, 4.508)	0.192
35–40 hours/week	90(25.4)	86(35.7)	1.026(0.629, 1.674)	0.918	0.977(0.544, 1.754)	0.938
41–48 hours/week	68(19.2)	42(17.4)	1.587(0.918, 2.745)	0.098	1.439(0.759, 2.729)	0.265
49–54 hours/week	48(13.6)	25(10.4)	1.882(1.011, 3.503)	0.046	1.383(0.669, 2.857)	0.382
≥55 hours/week	72(20.3)	26(10.8)	2.715(1.498, 4.919)	0.001	2.213(1.125, 4.355)	0.021
P for trend				0.001		0.022
OPA^a^						
None	51(14.4)	50(20.7)	1		1	
Heavy	34(9.6)	43(17.8)	0.775(0.427, 1.406)	0.402	0.674(0.336, 1.352)	0.267
Moderate	46(13.0)	43(17.8)	1.049(0.593, 1.855)	0.870	0.972(0.499, 1.896)	0.934
Light	66(18.6)	51(21.2)	1.269(0.743, 2.165)	0.383	1.302(0.695, 2.439)	0.411
Sedentary	157(44.4)	54(22.4)	2.850(1.733,4.689)	<0.001	2.794(1.526, 5.115)	0.001
P for trend				0.004		0.005

Abbreviations: OPA, occupational physical activity; CHD, coronary heart disease; OR, odds ratio; CI, confidence interval.

^a^ Qualitative variables were used to express as numbers and frequencies (%) tested by Pearson’s χ2, and all were statistically significant (P<0.05).

^b^ Adjustment for age, gender, body mass index, hypertension, diabetes mellitus, hyperlipidemia, smoking status, alcohol use, physical activity, and education.

Table 6 illustrates that a significant percentage (64.9%) of the employed sedentary population engaged in overtime work (>40 h/w). Therefore, the majority of the participants in sedentary occupations often worked overtime (P = 0.029).

**Table 6:** Distribution of worktime between sedentary and non-sedentary employed population.

	Sedentary(%)(n = 211)	Non- Sedentary (%)(n = 283)	P
Classification of worktime			0.029
<35 hours/week	15(7.1)	22(7.8)	
35–40 hours/week	59(30.0)	117(41.3)	
41–48 hours/week	51(24.2)	59(20.8)	
49–54 hours/week	37(17.5)	36(12.7)	
≥55 hours/week	49(23.2)	49(17.3)	

Categorical variables, expressed as numbers and frequencies (%), used Pearson’s χ2 for comparison.

## Discussion

Recently, overtime work and sedentary occupations have become dominant work trends and have shown associations with many healthy problems, particularly in relation to CHD[8, 10, 15, 19, 23, 25]. Here, we found that working long hours (especially over 55 h per week) and sedentary behavior at work each statistically significantly increased the risk of CHD. Moreover, we found that prolonged working hours in sedentary occupations increased the risk of CHD, regardless of engagement in exercise during leisure time.

Several studies, conducted in Asian countries and surrounding areas, have reported an association between working long hours and AMI, with the risk of AMI increasing as work time prolonged[3, 8, 32]. Similar results have been found for Europe and America. A British cohort study demonstrated that overtime work was an independent risk factor for CHD[33]. Compared with standard working time, working 3–4 h overtime per day was associated with a 1.56-fold increased risk of CHD. In addition, Mika Kivimäki found that working long hours, especially ≥55 h/w, was a risk factor for CHD and that employees who worked long hours had a 1.13-fold higher risk of CHD than those working standard hours[23]. These findings are in line with our current results indicating that working long hours negatively affects the incidence of CHD (OR = 2.213, 95%CI: 1.125, 4.355) with a significant dose-response relationship (P = 0.022). Meanwhile, we investigated the association between OPA and CHD risk and found an adverse relationship between sedentary occupation and CHD (OR = 2.794, 95%CI: 1.526, 5.115, P = 0.001) with a linear relationship (P for the trend = 0.005) after multivariate adjustment for exercise and other participant characteristics. Independent of engagement in exercise during leisure time, the lesser the OPA during working hours, the higher the incidence of CHD. A prospective cohort study examined the link between OPA and the occurrence of cardiovascular disease (CVD) in a Japanese population and concluded that low OPA is associated with high CVD-related mortality[8]. Other reports have indicated that sedentary behavior is associated with adverse health, including an increased risk for CVD, DM, cancer and all-cause mortality[27].

In the present study, the majority of the participants who worked overtime had sedentary behavior. Long-term work in a sedentary occupation increased the occurrence of CHD, regardless of engagement in physical activity during leisure time. The mechanisms of this association are still unclear, despite extensive investigation. Some studies have concluded that working overtime could increase the CHD risk factors of blood pressure, lipid levels and atherosclerosis. One study indicated that people who worked long hours had persistent activation of the sympathetic nervous system due to short rest time and incomplete recovery of the body[34], which could lead to long-term increases in blood pressure and serum lipid levels. Moreover, people who work long hours often did so under great pressure, and chronic stress increased the incidence of CHD by 40%-60%[35]. Long-term work stress caused the continuous contraction of blood vessels by activating the sympathetic nervous system and reducing NO production, thereby leading to myocardial ischemia[36]. More importantly, work stress could accelerate the rate of endothelial cell necrosis, resulting in impairment of endothelial function. This impairment could increase inflammatory factors level and reduce NO production[37], increasing the likelihood of atherosclerotic plaque formation as well as the risk of developing CHD. As for the molecular basis of the relationship between OPA and CHD, one explanation is the regulation of lipoprotein lipase (LPL), an enzyme inversely associated with CHD. This enzyme hydrolyzes lipoproteins and triglycerides in the vascular endothelium[38], and animal studies using rats have shown that its activity in inactive animals is reduced to only 10% of that in active animals, with the former group easily developing hyperlipidemia[39, 40]. It is interesting to note that the concentration of LPL messenger RNA (mRNA) does not decrease in inactive muscle, despite that LPL activity is reduced, whereas LPL mRNA expression increase after movement to reverse LPL activity, independent of movement intensity[27, 39, 41]. Based on this relationship, it is advisable that individuals experiencing long-term sedentary work conditions take frequent walks to balance lipid metabolism. Another hypothesis is that prolonged sitting decreases insulin activity due to the energy surplus created[42]. Previous studies have assumed that insulin signal transduction was disrupted by excess free fatty acids and amino acids, resulting in decreased insulin activity and insulin resistance[43]. Notably, disturbances of lipid and carbohydrate metabolism are risk factors of CHD.

Previous studies have highlighted the adverse effects of inadequate leisure-time exercise or total sitting time on CHD[18]. Based on these relationships, many studies have advocated increasing exercise participation after work to decrease CHD risk, which has been widely proven to be effective[29, 44]. Current recommendations from public health guidelines promote a minimum of 30 min/day of leisure-time physical activity with at least moderate-intensity 5 days/week[44]. Our team also previously investigated the relationship between engagement in sports-related physical activity after work and AMI, and we found that the activity had a protective function in a Chinese population[29]. However, although engagement in physical activity after work is beneficial for health, it might not entirely protect against the hazards associated with prolonged sitting[38, 45]. In addition to regular exercise, creating workplace regulations to address prolonged sedentary time is essential, as prolonged sitting is an independent risk factor for CHD. Decreasing sitting time by shortening or breaking up sedentary time at work is one potential way to prevent CHD. We advocate that people who must spend long hours sitting at work shorten their sedentary time by periodically moving away from the desk or taking a walk, whether or not they take part in physical exercise. Additional studies should focus on the effect of breaking up prolonged sitting time during work on CHD risk.

### Limitations of the study

This study had several limitations. First, our research used a retrospective design, which potentially led to recall bias. Second, all subjects were Chinese and enrolled from the same hospital, and the sample size was small; therefore, this study might not be representative of the general population. A large-scale, multi-center prospective study should be conducted in the future to overcome this limitation. Third, working time and OPA were both self-reported by the participants, which made it difficult to calculate the precise timing and quantity of active versus inactive behavior. Future intervention trials should use measurements such as a pedometer and accelerometer to obtain more quantitative information. Finally, uric acid and renal function were not evaluated as predictors of CHD, and future studies should take these parameters into consideration.

## Conclusion

Compared to non-employed people, individuals engaging in long working hours and sedentary behavior at work are at an increased risk of CHD. After adjusting for engagement in physical activity during leisure time, prolonged working hours in sedentary occupations increased the risk of CHD. Regardless of whether they exercise after work, we advocate that people who sit for long hours at work reduce their working hours and degree of sedentary behavior at work to help prevent CHD.

## Supporting information

S1 Table Characteristics of CHD and non-CHD groups. (DOCX)

S2 Table Univariate logistic regression for CHD risk factors. (DOCX)

S3 Table Multivariate logistic regression for CHD risk factors. (DOCX)

S4 Table Relationship between occupational characteristics and CHD. (DOCX)

S5 Table Relationship between worktime, OPA and the risk of CHD. (DOCX)

S6 Table Distribution of worktime between sedentary and non-sedentary employed population. (DOCX)

S1 Appendix Occupational Physical Activity Questionnaire (OPAQ). (DOCX)

S2 Appendix Job Content Questionnaire (JCQ) Scales. (DOCX)

S1 File STROBE checklist. (DOCX)

## Abbreviations

CHD: coronary heart disease
OPA: occupational physical activity
OR: odds ratio
CI: confidence interval
CAG: coronary angiography
OPAQ: occupational physical activity questionnaire
METs: metabolic equivalent tasks
BMI: body mass index
TC: total cholesterol
HDL: high-density lipoprotein
LDL: low-density lipoprotein
JCQ: Job Content Questionnaire
SPSS: Statistics Package for Social Sciences
AMI: acute myocardial infarction
CVD: cardiovascular disease
DM: diabetes mellitus
LPL: lipoprotein lipase
mRNA: messenger RNA.
