# Supplementary material for: Study on association of working hours and occupational physical activity with the occurrence of coronary heart disease in a Chinese population
Source: PLoS One. 2017 Oct 19;12(10):e0185598. doi: 10.1371/journal.pone.0185598 (PMC5648113; doi:10.1371/journal.pone.0185598)
Supplement: S1 Table — (DOCX) [file pone.0185598.s001.docx]

Table 1. Characteristics of CHD and non-CHD groups

| Characteristics | CHD(%)（n=354） | Non-CHD(%)（n=241） | P |
| --- | --- | --- | --- |
| Age | 55.28±7.01 | 51.94±8.55 | ＜0.001 |
| Male | 274(77.4) | 132(54.8) | ＜0.001 |
| BMI | 25.54±2.91 | 24.90±3.18 | 0.011 |
| Hypertension | 250(70.6) | 127(52.7) | ＜0.001 |
| Diabetes | 95(26.8) | 21(8.7) | ＜0.001 |
| Hyperlipidemia | 252(71.2) | 135(56.0) | ＜0.001 |
| Family history of CHD | 80(22.6) | 31(12.9) | 0.003 |
| Sports-related Physical activity | 53(15.0) | 53(22.0) | 0.028 |
| Smoking status |  |  | ＜0.001 |
| Never | 146(41.2) | 146(60.6) |  |
| Former | 52(14.7) | 23(9.5) |  |
| Current | 156(44.1) | 72(29.9) |  |
| Drinking status |  |  | 0.01 |
| Never | 177(50.0) | 160(66.4) |  |
| Former | 70(19.8) | 30(12.4) |  |
| Current | 107(30.2) | 51(21.2) |  |
| Education |  |  | 0.249 |
| Illiteracy | 18(5.1) | 17(7.1) |  |
| Primary | 54(15.3) | 26(10.8) |  |
| Middle | 98(27.7) | 81(33.6) |  |
| High | 113(31.9) | 68(28.2) |  |
| College | 71(20.1) | 49(20.3) |  |
| Employment status | 303(85.6) | 191(79.3) | 0.043 |

1. Abbreviations: CHD, coronary heart disease; BMI, body mass index.

2.Continuous values (age and BMI) were expressed as mean ± SD and Student’s t-test was used for comparison.

3.The rest were categorical variables expressed as numbers and frequencies (%), compared by Pearson’s χ2-test.
