# Supplementary material for: Study on association of working hours and occupational physical activity with the occurrence of coronary heart disease in a Chinese population
Source: PLoS One. 2017 Oct 19;12(10):e0185598. doi: 10.1371/journal.pone.0185598 (PMC5648113; doi:10.1371/journal.pone.0185598)
Supplement: S2 Table — (DOCX) [file pone.0185598.s002.docx]

Table 2. Univariate logistic regression for CHD risk factors

| Characteristic | All subjects | | |
| --- | --- | --- | --- |
|  | OR | 95%CI | P |
| Age | 1.057 | 1.034, 1.081 | ＜0.001 |
| Gender (Male=1, Female=2) | 0.354 | 0.248, 0.505 | ＜0.001 |
| BMI | 1.073 | 1.016, 1.134 | 0.012 |
| Hypertension (N=0, Y=1) | 2.196 | 1.563, 3.086 | ＜0.001 |
| Diabetes (N=0, Y=1) | 3.843 | 2.318, 6.371 | ＜0.001 |
| Hyperlipidemia (N=0, Y=1) | 1.940 | 1.377, 2.733 | ＜0.001 |
| Family history of CHD (N=0, Y=1) | 1.978 | 1.259, 3.108 | 0.003 |
| Sports-related Physical activity (N=0, Y=1) | 0.625 | 0.410, 0.953 | 0.029 |
| Smoking status |  |  |  |
| Never | 1 |  |  |
| Former | 2.261 | 1.315, 3.887 | 0.003 |
| Current | 2.167 | 1.510, 3.110 | ＜0.001 |
| Drinking status |  |  |  |
| Never | 1 |  |  |
| Former | 2.109 | 1.308, 3.402 | 0.002 |
| Current | 1.897 | 1.276, 2.818 | 0.002 |
| Education |  |  |  |
| Illiteracy | 1 |  |  |
| Primary | 1.962 | 0.871, 4.415 | 0.104 |
| Middle | 1.143 | 0.553, 2.360 | 0.719 |
| High | 1.569 | 0.758, 3.250 | 0.225 |
| College | 1.368 | 0.642, 2.915 | 0.416 |
| Employment status (N=0, Y=1) | 1.555 | 1.012, 2.391 | 0.044 |

Abbreviations: CHD, coronary heart disease; BMI, body mass index; OR, odds ratio; CI, confidence interval.
