# Supplementary material for: Study on association of working hours and occupational physical activity with the occurrence of coronary heart disease in a Chinese population
Source: PLoS One. 2017 Oct 19;12(10):e0185598. doi: 10.1371/journal.pone.0185598 (PMC5648113; doi:10.1371/journal.pone.0185598)
Supplement: S3 Table — (DOCX) [file pone.0185598.s003.docx]

Table 3. Multivariate logistic regression for CHD risk factors

| Characteristic | All subjects | | |
| --- | --- | --- | --- |
|  | OR | 95%CI | P |
| Age | 1.059 | 1.032, 1.087 | ＜0.001 |
| Gender (Male=1, Female=2) | 0.324 | 0.186, 0.565 | ＜0.001 |
| BMI | 1.053 | 0.989, 1.123 | 0.109 |
| Hypertension (N=0, Y=1) | 1.773 | 1.198, 2.624 | 0.004 |
| Diabetes (N=0, Y=1) | 3.447 | 1.985, 5.986 | ＜0.001 |
| Hyperlipidemia (N=0, Y=1) | 1.627 | 1.090, 2.431 | 0.017 |
| Family history of CHD (N=0, Y=1) | 1.878 | 1.126, 3.130 | 0.016 |
| Sports-related Physical activity (N=0, Y=1) | 0.585 | 0.361, 0.949 | 0.030 |
| Smoking status |  |  |  |
| Never | 1 |  |  |
| Former | 1.026 | 0.507, 2.077 | 0.944 |
| Current | 1.178 | 0.685, 2.025 | 0.554 |
| Drinking status |  |  |  |
| Never | 1 |  |  |
| Former | 1.216 | 0.677, 2.184 | 0.513 |
| Current | 1.010 | 0.602, 1.696 | 0.970 |
| Education |  |  |  |
| Illiteracy | 1 |  |  |
| Primary | 2.002 | 0.798, 5.024 | 0.139 |
| Middle | 0.864 | 0.372, 2.003 | 0.732 |
| High | 0.966 | 0.405, 2.302 | 0.938 |
| College | 0.839 | 0.338, 2.082 | 0.705 |
| Employment status (N=0, Y=1) | 1.396 | 0.834, 2.337 | 0.204 |

1. Abbreviations: CHD, coronary heart disease; BMI, body mass index; OR, odds ratio; CI, confidence interval.

2.Multivariate adjustment for age, gender, body mass index, hypertension, diabetes mellitus, hyperlipidemia, smoking status, alcohol use, physical activity, and education.
