# Supplementary material for: Study on association of working hours and occupational physical activity with the occurrence of coronary heart disease in a Chinese population
Source: PLoS One. 2017 Oct 19;12(10):e0185598. doi: 10.1371/journal.pone.0185598 (PMC5648113; doi:10.1371/journal.pone.0185598)
Supplement: S4 Table — (DOCX) [file pone.0185598.s004.docx]

Table 4. Relationship between occupational characteristics and CHD

| Characteristics | CHD  (n=354) | Non-CHD (n=241) | Unadjusted OR(95%CI) | P | Adjusted OR(95%CI) | P |
| --- | --- | --- | --- | --- | --- | --- |
| Employment status* |  |  |  |  |  |  |
| NO | 51 | 50 | 1 |  | 1 |  |
| Yes | 303 | 191 | 1.555(1.012, 2.391) | 0.044 | 1.396(0.834, 2.337) | 0.204 |
| Job Strain* |  |  |  |  |  |  |
| None | 51(14.4) | 50(20.7) | 1 |  | 1 |  |
| Low | 63(17.8) | 54(22.4) | 1.144(0.671, 1.949) | 0.621 | 1.054(0.571, 1.944) | 0.867 |
| Moderate | 123(34.7) | 96(39.8) | 1.256(0.783, 2.015) | 0.344 | 1.205(0.690, 2.104) | 0.513 |
| High | 117(33.1) | 41(17.0) | 2.798(1.650, 4.743) | ＜0.001 | 2.384(1.290, 4.405) | 0.006 |
| P for trend |  |  |  | ＜0.001 |  | 0.004 |
| Rest time* |  |  |  |  |  |  |
| None | 51(14.4) | 50(20.7) | 1 |  | 1 |  |
| ≤4 days/month | 194(54.8) | 128(53.1) | 1.486(0.948, 2.329) | 0.084 | 1.419(0.830, 2.427) | 0.201 |
| 5-8 days/month | 104(29.4) | 56(23.2) | 1.821(1.096, 3.025) | 0.021 | 1.505(0.819, 2.764) | 0.188 |
| ＞8 days/month | 5(1.4) | 7(2.9) | 0.700(0.208, 2.353) | 0.565 | 0.479(0.122, 1.882) | 0.291 |
| P for trend |  |  |  | 0.108 |  | 0.613 |
| Security of work* |  |  |  |  |  |  |
| None | 51(14.4) | 50(20.7) | 1 |  | 1 |  |
| Insecurity | 61(17.2) | 36(14.9) | 1.661 (0.942, 2.930) | 0.080 | 1.669(0.867, 3.212) | 0.125 |
| Security | 242(68.4) | 155(64.3) | 1.531 (0.987, 2.375) | 0.057 | 1.326(0.782, 2.249) | 0.294 |

1. Abbreviations: CHD, coronary heart disease; OR, odds ratio; CI, confidence interval.

2.* Qualitative variables were used to express as numbers and frequencies (%) tested by Pearson’s χ2, and both were statistically significant (P＜0.05).

3. Adjustment for age, gender, body mass index, hypertension, diabetes mellitus, hyperlipidemia, smoking status, alcohol use, physical activity, and education.
