# Supplementary material for: Study on association of working hours and occupational physical activity with the occurrence of coronary heart disease in a Chinese population
Source: PLoS One. 2017 Oct 19;12(10):e0185598. doi: 10.1371/journal.pone.0185598 (PMC5648113; doi:10.1371/journal.pone.0185598)
Supplement: S5 Table — (DOCX) [file pone.0185598.s005.docx]

Table 5. Relationship between worktime, OPA and the risk of CHD

| Characteristics | CHD  (n=354） | Non-CHD  (n=241) | Unadjusted OR(95%CI) | P | Adjusted OR(95%CI) | P |
| --- | --- | --- | --- | --- | --- | --- |
| Worktime* |  |  |  |  |  |  |
| None | 51(14.4) | 50(20.7) | 1 |  | 1 |  |
| ＜35 hours/week | 25(7.1) | 12(5.0) | 2.042(0.926, 4.506) | 0.077 | 1.825(0.739, 4.508) | 0.192 |
| 35-40 hours/week | 90(25.4) | 86(35.7) | 1.026(0.629, 1.674) | 0.918 | 0.977(0.544, 1.754) | 0.938 |
| 41-48 hours/week | 68(19.2) | 42(17.4) | 1.587(0.918, 2.745) | 0.098 | 1.439(0.759, 2.729) | 0.265 |
| 49-54 hours/week | 48(13.6) | 25(10.4) | 1.882(1.011, 3.503) | 0.046 | 1.383(0.669, 2.857) | 0.382 |
| ≥55 hours/week | 72(20.3) | 26(10.8) | 2.715(1.498, 4.919) | 0.001 | 2.213(1.125, 4.355) | 0.021 |
| P for trend |  |  |  | 0.001 |  | 0.022 |
| OPA* |  |  |  |  |  |  |
| None | 51(14.4) | 50(20.7) | 1 |  | 1 |  |
| Heavy | 34(9.6) | 43(17.8) | 0.775(0.427, 1.406) | 0.402 | 0.674(0.336, 1.352) | 0.267 |
| Moderate | 46(13.0) | 43(17.8) | 1.049(0.593, 1.855) | 0.870 | 0.972(0.499, 1.896) | 0.934 |
| Light | 66(18.6) | 51(21.2) | 1.269(0.743, 2.165) | 0.383 | 1.302(0.695, 2.439) | 0.411 |
| Sedentary | 157(44.4) | 54(22.4) | 2.850(1.733,4.689) | ＜0.001 | 2.794(1.526, 5.115) | 0.001 |
| P for trend |  |  |  | 0.004 |  | 0.005 |

1. Abbreviations: OPA, occupational physical activity; CHD, coronary heart disease; OR, odds ratio; CI, confidence interval.

2.* Qualitative variables were used to express as numbers and frequencies (%) tested by Pearson’s χ2, and all were statistically significant (P＜0.05).

3. Adjustment for age, gender, body mass index, hypertension, diabetes mellitus, hyperlipidemia, smoking status, alcohol use, physical activity, and education.
