# Supplementary material for: Study on association of working hours and occupational physical activity with the occurrence of coronary heart disease in a Chinese population
Source: PLoS One. 2017 Oct 19;12(10):e0185598. doi: 10.1371/journal.pone.0185598 (PMC5648113; doi:10.1371/journal.pone.0185598)
Supplement: S6 Table — (DOCX) [file pone.0185598.s006.docx]

Table 6. Distribution of worktime between sedentary and non-sedentary employed population

|  | Sedentary(%)（n=211） | Non- Sedentary (%)（n=283） | P |
| --- | --- | --- | --- |
| Classification of worktime |  |  | 0.029 |
| ＜35 hours/week | 15(7.1) | 22(7.8) |  |
| 35-40 hours/week | 59(30.0) | 117(41.3) |  |
| 41-48 hours/week | 51(24.2) | 59(20.8) |  |
| 49-54 hours/week | 37(17.5) | 36(12.7) |  |
| ≥55 hours/week | 49(23.2) | 49(17.3) |  |

Categorical variables, expressed as numbers and frequencies (%), used Pearson’s χ2 for comparison.
