# Supplementary material for: Study on association of working hours and occupational physical activity with the occurrence of coronary heart disease in a Chinese population
Source: PLoS One. 2017 Oct 19;12(10):e0185598. doi: 10.1371/journal.pone.0185598 (PMC5648113; doi:10.1371/journal.pone.0185598)
Supplement: S1 Appendix — (DOCX) [file pone.0185598.s007.docx]

**Appendix 1. Occupational Physical Activity Questionnaire (OPAQ)**

| **Occupational Physical Activity Questionnaire (OPAQ)** |
| --- |
| 1.How many hours per week do you usually work in your primary job? |
| 2.In a usual week, do you perform any sitting or standing doing work such as using a computer, desk work, using hand tools, light assembly, lab tech, or driving a car or truck while at work? |
| 3.In a usual week, how many hours do you do these sitting or standing activities at work? |
| 4.In a usual week, do you perform any walking at work as in the halls, between buildings, or in jobs like a postal carrier, waiter, or roving salesperson? |
| 5.In a usual week, how many hours do you walk at work? |
| 6.In a usual week, do you perform any heavy labor or use power tools during work such as moving furniture, carpentry, jackhammers, or using a shovel or pick? |
| 7.In a usual week, how many hours do you perform these heavy labor activities at work? |
