# Supplementary material for: Study on association of working hours and occupational physical activity with the occurrence of coronary heart disease in a Chinese population
Source: PLoS One. 2017 Oct 19;12(10):e0185598. doi: 10.1371/journal.pone.0185598 (PMC5648113; doi:10.1371/journal.pone.0185598)
Supplement: S2 Appendix — (DOCX) [file pone.0185598.s008.docx]

**Appendix 2. Job Content Questionnaire (JCQ) Scales**

| **Job Content Questionnaire (JCQ) Scales** | |
| --- | --- |
| Decision latitude (job control) | Psychological job demands |
| 1、learn new things | 1、work fast |
| 2、repetitive work | 2、work hard |
| 3、requires creative | 3、no excessive work |
| 4、high skill level | 4、enough time |
| 5、variety | 5、conflicting demands |
| 6、develop own abilities |  |
| 7、allow own decisions |  |
| 8、little decision of freedom |  |
| 9、lot of say |  |
