# Supplementary material for: Study on association of working hours and occupational physical activity with the occurrence of coronary heart disease in a Chinese population
Source: PLoS One. 2017 Oct 19;12(10):e0185598. doi: 10.1371/journal.pone.0185598 (PMC5648113; doi:10.1371/journal.pone.0185598)
Supplement: S1 File — (DOCX) [file pone.0185598.s009.docx]

STROBE Statement—checklist of items that should be included in reports of observational studies

|  | Item No. | Recommendation | Page  No. | Relevant text from manuscript |
| --- | --- | --- | --- | --- |
| **Title and abstract** | 1 | (*a*) Indicate the study’s design with a commonly used term in the title or the abstract | 1-2 | Coronary heart disease; Working hours; Occupational physical activity |
|  |  | (*b*) Provide in the abstract an informative and balanced summary of what was done and what was found | 1-2 | Long working hours and sedentary behavior at work were associated with high risk of CHD, |
| Introduction | | | |  |
| Background/rationale | 2 | Explain the scientific background and rationale for the investigation being reported | 3-4 | CHD is still the leading cause of death and disability across the world |
| Objectives | 3 | State specific objectives, including any prespecified hypotheses | 4 | To investigate the relationship between working hours and OPA with the occurrence of CHD in a Chinese population. |
| Methods | | | |  |
| Study design | 4 | Present key elements of study design early in the paper | 4-5 | This study was designed as a case-control study which enrolled 595 participants |
| Setting | 5 | Describe the setting, locations, and relevant dates, including periods of recruitment, exposure, follow-up, and data collection | 5-7 |  |
| Participants | 6 | (*a*) *Cohort study*—Give the eligibility criteria, and the sources and methods of selection of participants. Describe methods of follow-up  *Case-control study*—Give the eligibility criteria, and the sources and methods of case ascertainment and control selection. Give the rationale for the choice of cases and controls  *Cross-sectional study*—Give the eligibility criteria, and the sources and methods of selection of participants | 4-5 | Aged between 24 and 65; Undergo coronary angiography for the first time. The case group had at least one main coronary artery with >50% luminal diameter stenosis and the control group were free of CHD. |
|  |  | (*b*) *Cohort study*—For matched studies, give matching criteria and number of exposed and unexposed  *Case-control study*—For matched studies, give matching criteria and the number of controls per case | 5 | Aged between 24 and 65, and the control group 93 consisted of 241 subjects |
| Variables | 7 | Clearly define all outcomes, exposures, predictors, potential confounders, and effect modifiers. Give diagnostic criteria, if applicable | 6-7 | Occupational-related physical activity evaluation was based on occupational physical activity questionnaire. |
| Data sources/ measurement | 8* | For each variable of interest, give sources of data and details of methods of assessment (measurement). Describe comparability of assessment methods if there is more than one group | *5-6* | *the First Affiliated Hospital of Nanjing Medical 88 University* |
| Bias | 9 | Describe any efforts to address potential sources of bias | 8-9 | All data in this study were processed by the Statistics Package for Social Sciences |
| Study size | 10 | Explain how the study size was arrived at | 4-5 | This study enrolled 595 participants aged between 24 and 65 during the period of December 2015 to November 2016 in Nanjing, China. |

Continued on next page

| Quantitative variables | 11 | Explain how quantitative variables were handled in the analyses. If applicable, describe which groupings were chosen and why | 8 | variance analysis |
| --- | --- | --- | --- | --- |
| Statistical methods | 12 | (*a*) Describe all statistical methods, including those used to control for confounding | 8 | Univariate and multiple logistic regression analyses |
|  |  | (*b*) Describe any methods used to examine subgroups and interactions | 8 | logistic regression analyses |
|  |  | (*c*) Explain how missing data were addressed | 6 | excluded from the study |
|  |  | (*d*) *Cohort study*—If applicable, explain how loss to follow-up was addressed  *Case-control study*—If applicable, explain how matching of cases and controls was addressed  *Cross-sectional study*—If applicable, describe analytical methods taking account of sampling strategy | 11 | sampling survey |
|  |  | (*e*) Describe any sensitivity analyses | 8 | SPSS |
| Results | | | | |
| Participants | 13* | (a) Report numbers of individuals at each stage of study—eg numbers potentially eligible, examined for eligibility, confirmed eligible, included in the study, completing follow-up, and analysed | 9-11 | logistic regression analysis |
|  |  | (b) Give reasons for non-participation at each stage | 9-11 | logistic regression analysis |
|  |  | (c) Consider use of a flow diagram | No |  |
| Descriptive data | 14* | (a) Give characteristics of study participants (eg demographic, clinical, social) and information on exposures and potential confounders | 9 | Table 1 presented the baseline characteristics of all the participants in the study. |
|  |  | (b) Indicate number of participants with missing data for each variable of interest |  |  |
|  |  | (c) *Cohort study*—Summarise follow-up time (eg, average and total amount) |  |  |
| Outcome data | 15* | *Cohort study*—Report numbers of outcome events or summary measures over time |  |  |
|  |  | *Case-control study—*Report numbers in each exposure category, or summary measures of exposure | *10-11* | *The distribution of long working hours was higher in case group than in control group. The lesser the OPA during working, the higher the incidence of CHD.* |
|  |  | *Cross-sectional study—*Report numbers of outcome events or summary measures |  |  |
| Main results | 16 | (*a*) Give unadjusted estimates and, if applicable, confounder-adjusted estimates and their precision (eg, 95% confidence interval). Make clear which confounders were adjusted for and why they were included | 9-11 | Working more than 55 h/w had an increased risk of CHD with a crude OR of 2.715 (95% CI: 1.498, 4.919, P=0.001), and an adjusted OR of 2.213 (95% CI: 1.125, 4.355, P=0.021) after multivariate adjustment in age, gender, hypertension, diabetes, hyperlipidemia, family history of CHD and sports-related physical activity. |
|  |  | (*b*) Report category boundaries when continuous variables were categorized | 6 | We divided working time into several groups: ＜35 h, 35–40 h, 41–48 h, 49–54 h, and ≥55 h per week. |
|  |  | (*c*) If relevant, consider translating estimates of relative risk into absolute risk for a meaningful time period | No |  |

Continued on next page

| Other analyses | 17 | Report other analyses done—eg analyses of subgroups and interactions, and sensitivity analyses | 10-11 | Table 4 described the relationship between occupational characteristics and CHD. |
| --- | --- | --- | --- | --- |
| Discussion | | | | |
| Key results | 18 | Summarise key results with reference to study objectives | 12 | Working long hours  245 (especially longer than 55h per week) and sedentary behavior at work had negative effects on the occurrence and increased the risk of CHD |
| Limitations | 19 | Discuss limitations of the study, taking into account sources of potential bias or imprecision. Discuss both direction and magnitude of any potential bias | 16 | Potential recall bias; might not be representative of the general population; imprecise calculation. |
| Interpretation | 20 | Give a cautious overall interpretation of results considering objectives, limitations, multiplicity of analyses, results from similar studies, and other relevant evidence | 17 | For people who sit for long hours at work, whether they exercised after work or not, we advocated them to reduce their working hours and degree of sedentary behavior at work for the prevention of CHD. |
| Generalisability | 21 | Discuss the generalisability (external validity) of the study results | 17 | Study on the risk factors of cardiovascular disease. |
| Other information | |  | | |
| Funding | 22 | Give the source of funding and the role of the funders for the present study and, if applicable, for the original study on which the present article is based | No |  |

*Give information separately for cases and controls in case-control studies and, if applicable, for exposed and unexposed groups in cohort and cross-sectional studies.

**Note:** An Explanation and Elaboration article discusses each checklist item and gives methodological background and published examples of transparent reporting. The STROBE checklist is best used in conjunction with this article (freely available on the Web sites of PLoS Medicine at http://www.plosmedicine.org/, Annals of Internal Medicine at http://www.annals.org/, and Epidemiology at http://www.epidem.com/). Information on the STROBE Initiative is available at www.strobe-statement.org.
